# Case Report: Severe form of hemolytic-uremic syndrome with multiple organ failure in a child: a case report

**DOI:** 10.12688/f1000research.2546.2

**Published:** 2014-06-20

**Authors:** Dino Mijatovic, Ana Blagaic, Zeljko Zupan

**Affiliations:** 1 Department of Anesthesiology and Critical Care, University Hospital Rijeka, Rijeka, Croatia

## Abstract

**Introduction:** Hemolytic-uremic syndrome (HUS) is a leading cause of acute renal failure in infants and young children. It is traditionally defined as a triad of acute renal failure, hemolytic anemia and thrombocytopenia that occur within a week after prodromal hemorrhagic enterocolitis. Severe cases can also be presented by acute respiratory distress syndrome (ARDS), toxic megacolon with ileus, pancreatitis, central nervous system (CNS) disorders and multiple organ failure (MOF).

**Case presentation:** A previously healthy 4-year old Caucasian girl developed acute renal failure, thrombocytopenia and hemolytic anemia following a short episode of abdominal pain and bloody diarrhea. By the end of the first week the diagnosis of the typical HUS was established. During the second week the disease progressed into MOF that included ileus, pancreatitis, hepatitis, coma and ARDS, accompanied by hemodynamic instability and extreme leukocytosis. Nonetheless, the girl made a complete recovery after one month of the disease. She was successfully treated in the intensive care unit and significant improvement was noticed after plasmapheresis and continuous veno-venous hemodialysis.

**Conclusions:** Early start of plasmapheresis and meticulous supportive treatment in the intensive care unit, including renal placement therapy, may be the therapy of choice in severe cases of HUS presented by MOF. Monitoring of prognostic factors is important for early performance of appropriate diagnostic and therapeutical interventions.

## Introduction

Hemolytic-uremic syndrome (HUS) can be classified as typical, usually when provoked by Shiga-toxin (STx) produced by enterohemorrhagic *Escherichia coli* serotype H157:O7, or atypical, when triggered by other microbes’ antigens and toxins ^1^. The overall incidence of STx-HUS is estimated to be 2.1 per 100 000 people, with a peak incidence in children younger than 5 years (6.1 per 100 000) ^1^. Approximately two-thirds of patients have to be dialyzed ^2^. Clinical manifestations of HUS are the consequence of severe systemic inflammation and immune reactions that lead to thrombotic microangiopathy in susceptible organs. In typical HUS, those reactions are initiated by binding of STx to the endothelial membrane-bound Gb3 receptor and subsequent activation of platelets and leukocytes ^3^, while in atypical HUS, those reactions are mediated by excessive activation of complement. Clinical manifestations can overlap with another similar syndrome – thrombotic thrombocytopenic purpura (TTP) ^5^. Typical HUS has better prognosis in terms of morbidity and mortality than atypical and severe forms of HUS complicated by multiple organ failure (MOF) ^1, 4^.

## Case description

**Figure 1.**
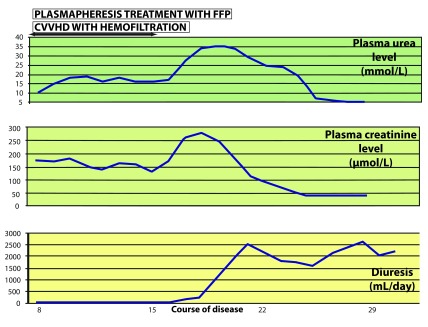
Gradual recovery of renal function. Plasma creatinine and urea levels and urine output during the disease course and applied treatment show significant improvement of renal function by the end of CVVHD and plasmapheresis and normal function is restored in the 4th week of disease without the evidence of permanent damage.

In 2012, a 4-year old Caucasian girl, on holiday with her parents, was brought to University Hospital Rijeka emergency department with abdominal cramps, vomiting and bloody diarrhea that had started 24 hours before her admission. Gastroenterocolitis was initially suspected, but as the abdominal pain intensified, an explorative laparotomy was performed on the third day of disease to exclude perforated appendicitis, which the surgeon eventually did not confirm. On the first postoperative day she appeared confused, dyspnoic, hypotensive, pale, oliguric and her laboratory findings were: hemoglobin (Hb) 60 g/L, hematocrit (Hct) 19%, platelet count 43×10 ^9^/L, white blood cell (WBC) count 17×10 ^9^/L, urea 16.6 mmol/L, creatinine 308 μmol/L, creatine kinase 971 U/L, aspartate aminotransferase (AST) 317 U/L, alanine transaminase (ALT) 276 U/L, C-reactive protein (CRP) 175.2 mg/L, lactate dehydrogenase (LDH) 3856 U/L, albumins 19 g/L, complement component 3 (C3) 0.5 g/L, complement component 4 (C4) 0.08 g/L, degree of *in vivo* hemolysis 50 mg/L, pH 7.265, pCO _2_ 3.64 kPa, pO _2_ 9.2 kPa, HCO _3_ 12 mmol/l, SBE -13.1 mmol/L. She was immediately admitted to intensive care where she was analgosedated with 6 mg of midazolam and 200 mcg of fentanyl, intubated with an uncuffed orotracheal tubus and invasive mechanical ventilation was started. During the first week of disease she received multiple transfusions of packed red blood cells, fresh frozen plasma and platelets as continuous adrenergic and diuretic support was established with 5–8 mcg/kg/min of dopamine and 50 mg/24 h of furosemide, respectively. After additional laboratory tests, typical HUS caused by reaction to *E. coli* toxin was diagnosed. By the end of the first week of disease her medical condition had not improved and she became anuric, accompanied by combined metabolic and respiratory acidosis, pulmonary edema, bilateral pleural and pericardial effusions, ascites and persistent anemia and thrombocytopenia. Continuous veno-venous hemodiafiltration (CVVHDF) as well as plasmapheresis were indicated. Access was established via an intravascular catheter placed through the right internal jugular vein guided by ultrasound. CVVHDF was performed for 12 days. Characteristics of dialysis included a dialyzer membrane surface area of 0.8 m ^2^, solution containing bicarbonate (multiBic 2 mmol/L potassium solution for haemofiltration; Fresenius Medical Care Deutschland GmbH) and a blood flow rate of 3–6 ml/kg/min. The system was anticoagulated with 100 IU/kg of unfractionated heparin each day. An additional blood flow rate for ultrafiltration was 0.5–2 ml/kg/h. The renal replacement therapy was only stopped for 3 hours a day in order to carry out plasmapheresis which was also initiated on the 8th day of disease. This lasted for 10 days, for 2 hours each day and 40 ml/kg of fresh frozen plasma was used per day. During this period continuous analgosedation with 0.15 mg/kg/h midazolam and 0.075 mg/kg/h morphine, as well as invasive mechanical ventilation were maintained. Continuous adrenergic support was stopped after the 6th day of disease. After 12 days of renal placement therapy, diuresis started to improve while serum creatinine and urea started to decrease. Despite the polyuria during the initial phase of recovery of renal function, because of the still significant fluid retention, we established negative fluid balance by continuous infusion of furosemid. Renal function had completely recovered by the 25th day of disease ( Figure 1). We have reason to believe that the blood levels of creatinine and urea at the time that the CVVHDF was started were falsely lower because of hemodilution caused by the body’s extreme retention of extracellular water.

During the course of plasmapheresis a marked recovery of the platelet count and hemoglobin level was noticed, as well as a significant decline of LDH levels and the degree of *in vivo* hemolysis. After termination of plasmapheresis, an additional time was needed for restoration of platelets and normal erythrocytes to optimal levels because of their biological cell cycle. We were restrictive throughout in the transfusion of packed red cells, aiming for a target hemoglobin level of 70–80 g/L.

**Figure 2.**
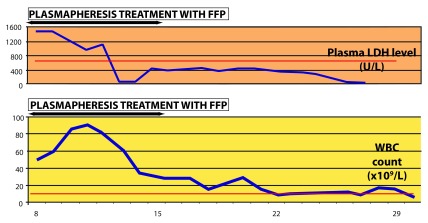
Extreme leukocytosis during the second week of disease indicated a high intensity of systemic inflammation, as the WBC correlated with LDH plasma levels. Plasmapheresis enabled the clearance of proinflammatory factors as the WBC and LDH level normalized during and after this course of treatment. Red lines denote upper reference limit in our laboratory - for plasma LDH 485 IU/L and WBC 10×10 ^9^/L.

The most striking laboratory finding during the first two weeks of disease was extreme leukocytosis which persisted during the second week of disease; the highest WBC count was 94×10 ^9^/L. As no other signs of systemic bacterial infection or invasive mycosis were noticed, we believe that leukocytosis was provoked by the systemic inflammatory response to toxemia. We observed the gradual decrease in the WBC count after the initiation of plasmapheresis and its subsequent normalization by the end of the third week of disease. This trend was correlated with the values of LDH plasma levels ( Figure 2).

**Figure 3.**
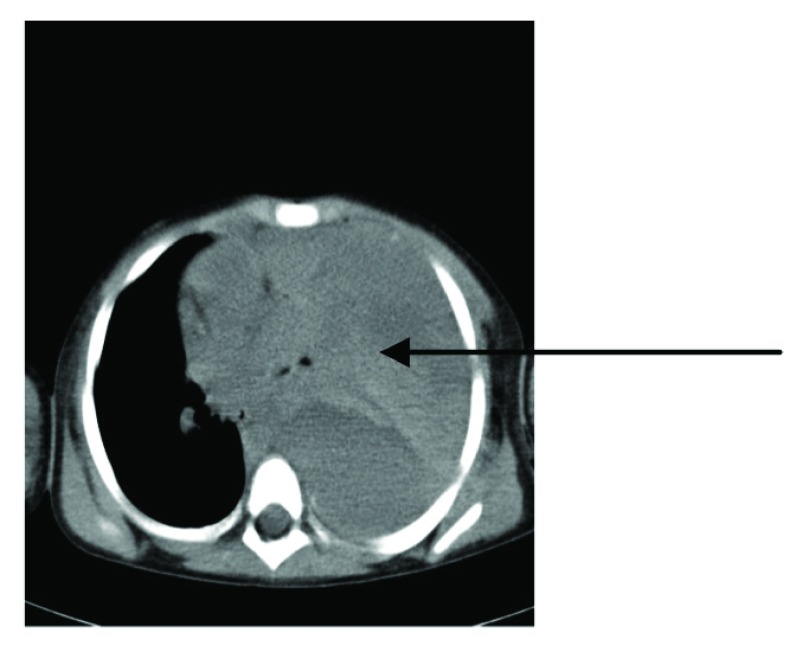
CT scan of thorax recorded on the 15th day of disease: massive left-sided hemothorax with atelectatic lung (arrow) that eventually required surgical treatment, as well as a careful strategy of protective invasive mechanical ventilation.

Bilateral pleural effusions needed evacuation by thoracocentesis on two occasions, on the 8th and 13th day of disease. A CT scan of the thorax performed on teh 15th day of disease revealed a massive left-sided hemothorax complicated by compressive and obstructive atelectasis of the left lung ( Figure 3). The hemothorax had to be surgically drained on the 16th day of disease. During the second week of disease we confirmed acute respiratory distress syndrome (ARDS) according to PaO _2_ to FiO _2_ ratio and evidence of hepatization of the lungs on serial ultrasound scans. Because of the pulmonary manifestations of the disease, a protective ventilation strategy using was employed. We mostly used airway pressure release ventilation (APRV) and biphasic positive airway pressure (BIPAP) modes on a Dräger Evita XL mechanical ventilator and frequently applied lung recruitment with the positive end-expiratory pressure (PEEP) technique monitored by ultrasound. Respiratory function completely recovered by the end of the third week of disease without apparent permanent damage and the girl was successfully extubated on the 24th day of disease.

**Figure 4.**
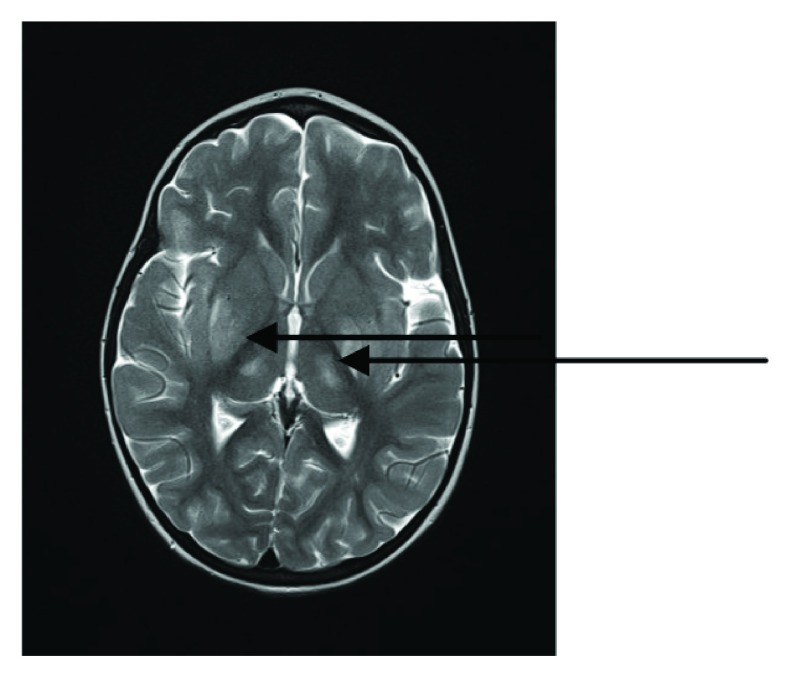
MRI scan of brain recorded on the 12th day of disease: bilateral hyperintensity in lateral thalamic regions (arrows).

We ceased the analgosedation on the 12th day of disease in order to evaluate the neurological status of the girl. She appeared comatose, estimated as GCS 4 (E2V1M1) on the Glasgow Coma Scale with flaccid tetraparesis, rotatory nystagmus and symmetrical mydriatic pupils with slow response to light stimulation. An MRI scan of the brain recorded on the 12th day of disease revealed areas of raised signal in the pons, bilaterally in lateral parts of the thalamus ( Figure 4), as well as in the right external capsule and the left internal capsule. These findings were analogous with lesions already described in literature believed to be the result of microvascular damage ^8^. Analgosedation was reinstated and afterwards periodically discontinued every few days for approximately 6 hours in order to examine the girl’s clinical neurological status. After the 18th day her level of consciousness and neurological status started to gradually improve and we decided to definitively cease analgosedation 7 days later. No permanent neurological damage was recorded.

**Figure 5.**
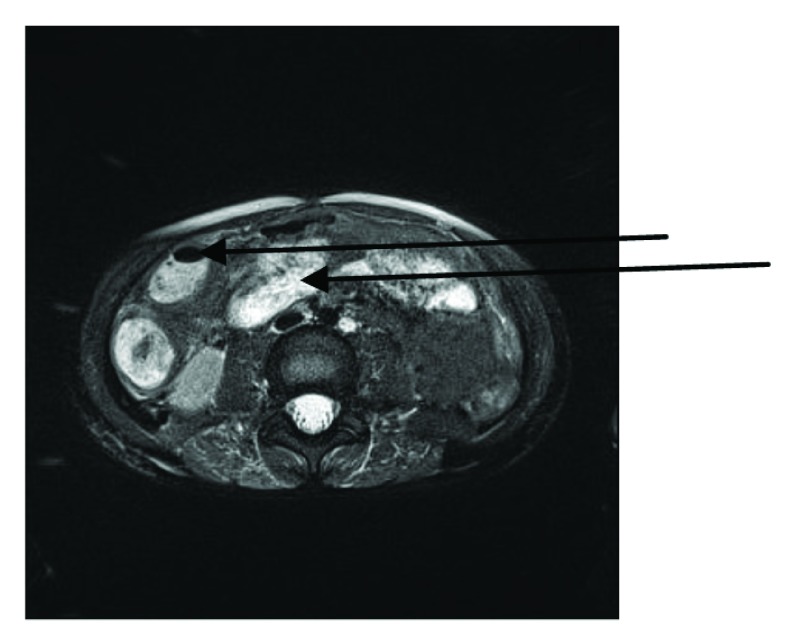
MRI scan of abdomen obtained on the 12th day of disease: edematous jejunal wall and distension with air fluid level in caecum (arrows).

We were concerned by severe gastric retention, absent or silent peristalsis and constipation with episodical soft and rare stools during the second week of disease. An MRI of the abdomen performed on the 12th day of disease showed an edematous intestinal wall and distension of the jejunum, ileum and colon ascendens with several air fluid levels ( Figure 5). Laboratory findings displayed elevated serum transaminases, total and direct bilirubin and amylase as well as hypoalbuminemia. After prodromal gastroenterocolitis, paralytic ileus obviously evolved and was persistent throughout the second and third week of disease. Therefore, all necessary fluid, macronutrients and micronutrients were completely delivered parenterally via a central venous catheter from the start of disease. In combination with already initiated parenteral nutrition, we carefully started enteral feeding by the end of the second week of disease. As no signs of intraabdominal compartment syndrome, perforation or peritonitis were registered, we wanted to reestablish gastrointestinal function conservatively. Therefore, we applied continuous infusion of 6–10 mg/24h of metoclopramid and intermittent rectal suppositories of 2.5–5 mg of bisacodyl for the next two weeks. We also progressively increased the contribution of the enteral input as soon as peristalsis started to improve. Bowel function slowly recovered by the beginning of the fourth week of disease.

As the patient and her family had been on holiday at the time of admission, an in-person follow up was not possible. However, the authors made contact with the family in 2013 and the parents reported that their daughter was perfectly healthy and without apparent consequences.

## Discussion and conclusion

Here we presented the case of a 4-year old girl who developed a severe form of typical HUS which initially presented classically, but rapidly progressed into MOF which severely affected the central nervous system, renal, gastrointestinal and cardiovascular function, and induced ARDS and hematological disorders.

Diarrhoea-associated HUS is the most severe clinical manifestation of infection with Shiga toxin-producing *Escherichia coli* and is more common in children ^1^. Pathogenesis of the syndrome is based on the reaction of the innate immune system to toxemia ^3^. Characteristic features of the syndrome are hemorrhagic enterocolitis, hemolytic anemia, thrombocytopenia and acute renal failure, but some patients develop more unusual manifestations that potentially lead to MOF and increase mortality ^4^. Even though 70% of children with HUS recover without permanent health consequences, 2–5% of patients die in the acute phase ^1, 2^.

Among less usual manifestations, CNS involvement is the most frequent and one that significantly increases morbidity and mortality ^6^. Neurological signs range from epileptic seizures to reduced consciousness level and focal motor deficits ^1, 7^, but are mostly the result of temporary dysfunction rather than irreversible damage. Brain MRI is the diagnostic method of choice when analyzing possible CNS lesions and usually reveals a pattern of symmetrical hyperintensities in basal ganglia and the thalamus ^8^.

The gastrointestinal and hepatobiliary tract may be affected from the esophagus to the perianal area and possible disorders include ileus, intussusception, bowel distension, perforation, necrosis, toxic megacolon, intestinal stricture, rectal prolapse, hepatocellular cholestasis and pancreatitis, which may lead to diabetes mellitus ^4, 9^.

Cardiovascular instability can be a consequence of tachyarrhythmia caused by HUS, electrolyte imbalance and toxic myocarditis with subsequent dilatative cardiomyopathy ^4, 6^.

The mainstay of treatment is supportive and includes control of fluid and electrolyte balance, optimal enteral and parenteral nutrition, use of hemodialysis (required in approximately two-thirds of patients ^2^), control of hemodynamic stability and judicious transfusion of blood derivatives. Plasmapheresis with fresh frozen plasma is reserved for the most severe cases ^10^, although it was proven ineffective in some recent controlled clinical trials ^11^. Platelet transfusions are avoided and limited for control of active bleeding, considering some studies have suggested it could contribute to the microthrombosis and worsen outcome ^4^. Use of antibiotics is controversial and should be avoided, since some studies have shown them to be harmful as possible triggers for the development of HUS in patients with enterohemorrhagic *E. coli* infection ^1^.

Already known predictors of poor outcome, including death and chronic renal and lung disease, are prolonged oliguria or anuria, need for hemodialysis, neurological impairment, persistent leukocytosis > 20×10 ^9^/L, hematocrit < 23% on admission and severe dysfunction of the gastrointestinal system ^6, 11^. The recorded extreme leukocytosis in this case was related to the particularly high activity of systemic inflammation as a result of toxemia rather than infection. It could be an indicator of HUS complicated with MOF and, therefore, one of the main negative prognostic factors ^2, 3, 6^. Despite the fact that all these predictors were recognized in the acute stage of disease, our patient fully recovered without any apparent sequelae during the follow-up period of one year. This case indicates that early diagnosis, thorough supportive treatment, including renal replacement therapy and early plasmapheresis, are crucial interventions for favorable outcomes in severe cases of typical HUS presented by MOF.

## Consent

Written informed consent for publication of this case report and corresponding images was obtained from the patient’s parents.

## References

[1] Noris M Remuzzi G: Hemolytic uremic syndrome. *J Am Soc Nephrol.* 2005;16(4):1035–50 10.1681/ASN.2004100861 15728781

[2] Gerber A Karch H Allerberger F: Clinical course and the role of Shiga toxin-producing Escherichia coli infection in the hemolytic-uremic syndrome in pediatric patients, 1997–2000, in Germany and Austria: a prospective study. *J Infect Dis.* 2002;186(4):493–500 10.1086/341940 12195376

[3] Exeni RA Fernández GC Palermo MS: Role of polymorphonuclear leukocytes in the pathophysiology of typical hemolytic uremic syndrome. *ScientificWorldJournal.* 2007;7:1155–64 10.1100/tsw.2007.172 17694250 PMC5901139

[4] Scheiring J Andreoli SP Zimmerhackl LB: Treatment and outcome of Shiga-toxin-associated hemolytic uremic syndrome (HUS). *Pediatr Nephrol.* 2008;23(10):1749–60 10.1007/s00467-008-0935-6 18704506 PMC6901419

[5] George JN Al-Nouri ZL: Diagnostic and therapeutic challenges in the thrombotic thrombocytopenic purpura and hemolytic uremic syndromes. *Hematology Am Soc Hematol Educ Program.* 2012;2012(1):604–609 10.1182/asheducation-2012.1.604 23233641

[6] Oakes RS Siegler RL McReynolds MA: Predictors of fatality in postdiarrheal hemolytic uremic syndrome. *Pediatrics.* 2006;117(5):1656–62 10.1542/peds.2005-0785 16651320

[7] Nathanson S Kwon T Elmaleh M: Acute neurological involvement in diarrhea-associated hemolytic uremic syndrome. *Clin J Am Soc Nephrol.* 2010;5(7):1218–28 10.2215/CJN.08921209 20498239 PMC2893076

[8] Steinborn M Leiz S Rüdisser K: CT and MRI in haemolytic uraemic syndrome with central nervous system involvement: distribution of lesions and prognostic value of imaging findings. *Pediatr Radiol.* 2004;34(10):805–10 10.1007/s00247-004-1289-2 15378218

[9] Riley MR Lee KK: Escherichia coli O157:H7–associated hemolytic uremic syndrome and acute hepatocellular cholestasis: a case report. *J Pediatr Gastroenterol Nutr.* 2004;38(3):352–354 15076639 10.1097/00005176-200403000-00022

[10] Bambauer R Latza R Schiel R: Therapeutic apheresis in the treatment of hemolytic uremic syndrome in view of pathophysiological aspects. *Ther Apher Dial.* 2011;15(1):10–19 10.1111/j.1744-9987.2010.00903.x 21272247

[11] Garg AX Suri RS Barrowman N: Long-term renal prognosis of diarrhea-associated hemolytic uremic syndrome: A systematic review, meta-analysis, and meta-regression. *JAMA.* 2003;290(10):1360–70 10.1001/jama.290.10.1360 12966129

