# Supplementary material for: Case Report: Severe form of hemolytic-uremic syndrome with multiple organ failure in a child: a case report
Source: F1000Res. 2014 Jun 20;3:79. Originally published 2014 Mar 27. [Version 2] doi: 10.12688/f1000research.2546.2 (PMC4097360; doi:10.12688/f1000research.2546.2)
Supplement: Peer review history

# Peer review: Referee response for version 2

**DOI:** 10.5256/f1000research.4879.r5252

**Published:** 2014-06-27

**Authors:** Grant Luxton

**Affiliations:** 1 Prince of Wales Clinical School, Faculty of Medicine, University of New South Wales, Sydney, NSW, Australia

The changes are minor and I have no issue with them.

I have read this submission. I believe that I have an appropriate level of expertise to confirm that it is of an acceptable scientific standard.

# Peer review: Referee response for version 2

**DOI:** 10.5256/f1000research.4879.r5239

**Published:** 2014-06-25

**Authors:** Zeljko Bosnjak

**Affiliations:** 1 Anesthesiology Research, Medical College of Wisconsin, Milwaukee, WI, USA

I have no further comments.

I have read this submission. I believe that I have an appropriate level of expertise to confirm that it is of an acceptable scientific standard.

# Peer review: Referee response for version 1

**DOI:** 10.5256/f1000research.2762.r4457

**Published:** 2014-04-30

**Authors:** Grant Luxton

**Affiliations:** 1 Prince of Wales Clinical School, Faculty of Medicine, University of New South Wales, Sydney, NSW, Australia

This is a case report documenting the response to treatment with best supportive care including dialysis as well as plasmapheresis in a four year old child with "typical" haemolytic uraemic syndrome. As the authors point out, trial evidence is not supportive of plasmapheresis for this condition however there may be situations where it may be of benefit - especially in a patient not responding to best supportive care. I have no issues with the case report and believe it is worth indexing to remind us that plasma exchange should still be considered in selected patients with this disease. With the potential availabilty of eculizumab however, the role of plasmapheresis may become even more limited.

I have read this submission. I believe that I have an appropriate level of expertise to confirm that it is of an acceptable scientific standard.

# Response

**Published:** 2014-06-04

**Authors:** Dino Mijatovic

**Affiliations:** University Hospital Rijeka, Croatia

I welcome your comment! We (all the authors) discussed the potential use of eculizumab during the early course of the disease in the second week. However, we didn't have any experience with it and it seemed to be very expensive treatment. Also, we found more data in the recent literature confirming the efficacy of this drug in atypical cases of HUS, rather than typical. Therefore, we decided to initiate the transfusion treatment with full awareness and precautions regarding its potential side-effects, and to leave the eculizumab as the second-line therapy.

# Peer review: Referee response for version 1

**DOI:** 10.5256/f1000research.2762.r4458

**Published:** 2014-04-17

**Authors:** Zeljko Bosnjak

**Affiliations:** 1 Anesthesiology Research, Medical College of Wisconsin, Milwaukee, WI, USA

The authors report a very interesting but extremely difficult case report that many will find quite helpful. I have few minor suggestions:

 

- Abstract (case presentation): rewrite the second sentence.

- Figures 1 and 2: Could easily be combined into one figure, with single labeling on the top, and placing descriptions of the parameters on top of each tracing. Also the units of diuresis should be changed to per day instead. For plasma creatinine, include the micro symbol.
-  Figures 3-5: Perhaps white arrows would be a better choice.

I have read this submission. I believe that I have an appropriate level of expertise to confirm that it is of an acceptable scientific standard.

# Response

**Published:** 2014-06-11

**Authors:** Dino Mijatovic

**Affiliations:** University Hospital Rijeka, Croatia

Dear Professor Bošnjak,

thank you very much for your comment! Unfortunately, we weren't able to write the response since we have been extremely occupied with organization of the national congress. Anyway, we found ourselves very grateful for your meticulous and thoughtful analysis. We decided to rewrite the second sentence in Case presentation. We also made proper corrections regarding the units of diuresis and plasma creatinine. We have still not combined figures 1 and 2 into one as we want to stress the extreme leukocytosis and its recovery during the PAF treatment, the process that was parallel to the monitored activity of LDH, which is the biomarker of this disease. We believe that this pattern becomes more appreciated when it is stressed in the figure with one or two diagrams. In the uniform picture it would be among 4 different diagrams.

We have left black arrows for figures 3-5 because their tops are a  greater contrast to the density of the object on the scan.
